# Supplementary material for: Pathological Mechanisms in Sjögren’s Disease Likely Involve the ADP-Ribosyl Cyclase Family Members: CD38 and CD157
Source: Int J Mol Sci. 2025 Nov 28;26(23):11544. doi: 10.3390/ijms262311544 (PMC12692564; doi:10.3390/ijms262311544)
Supplement: Supplementary file 1 [file ijms-26-11544-s001.zip › ijms-3943017-supplementary.pdf]

Supplementary Material

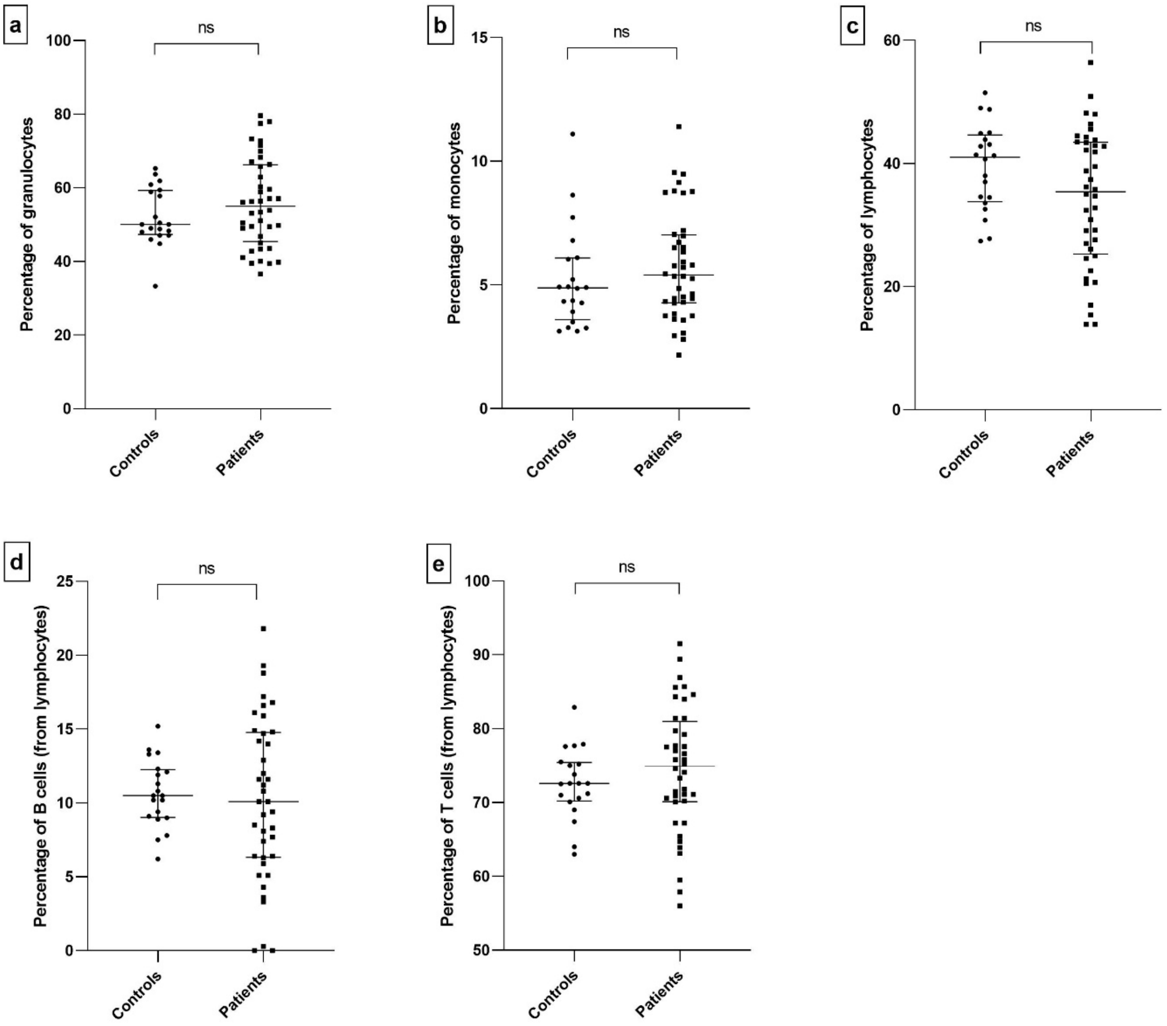

Figure S1. Percentages of individual leukocyte populations and subpopulations in patients and controls. (Sub)Populations were distinguished based on their distinct side-scattered light and forward-scattered light characteristics, as well as the differential expression of cell-surface antigens (CD14, CD3, and CD19). Comparison of granulocytes (p = 0.309) (**a**), monocytes (p = 0.261) (**b**), lymphocytes (p = 0.082) (**c**), B cells (p = 0.685) (**d**), and T cells (p = 0.284) (**e**). Statistical significance was indicated as described in Figure 1 ($p \leq 0.05$ (*), $p \leq 0.01$ (**), $p \leq 0.001$ (***), and non-significant differences ($p > 0.05$) as ns).

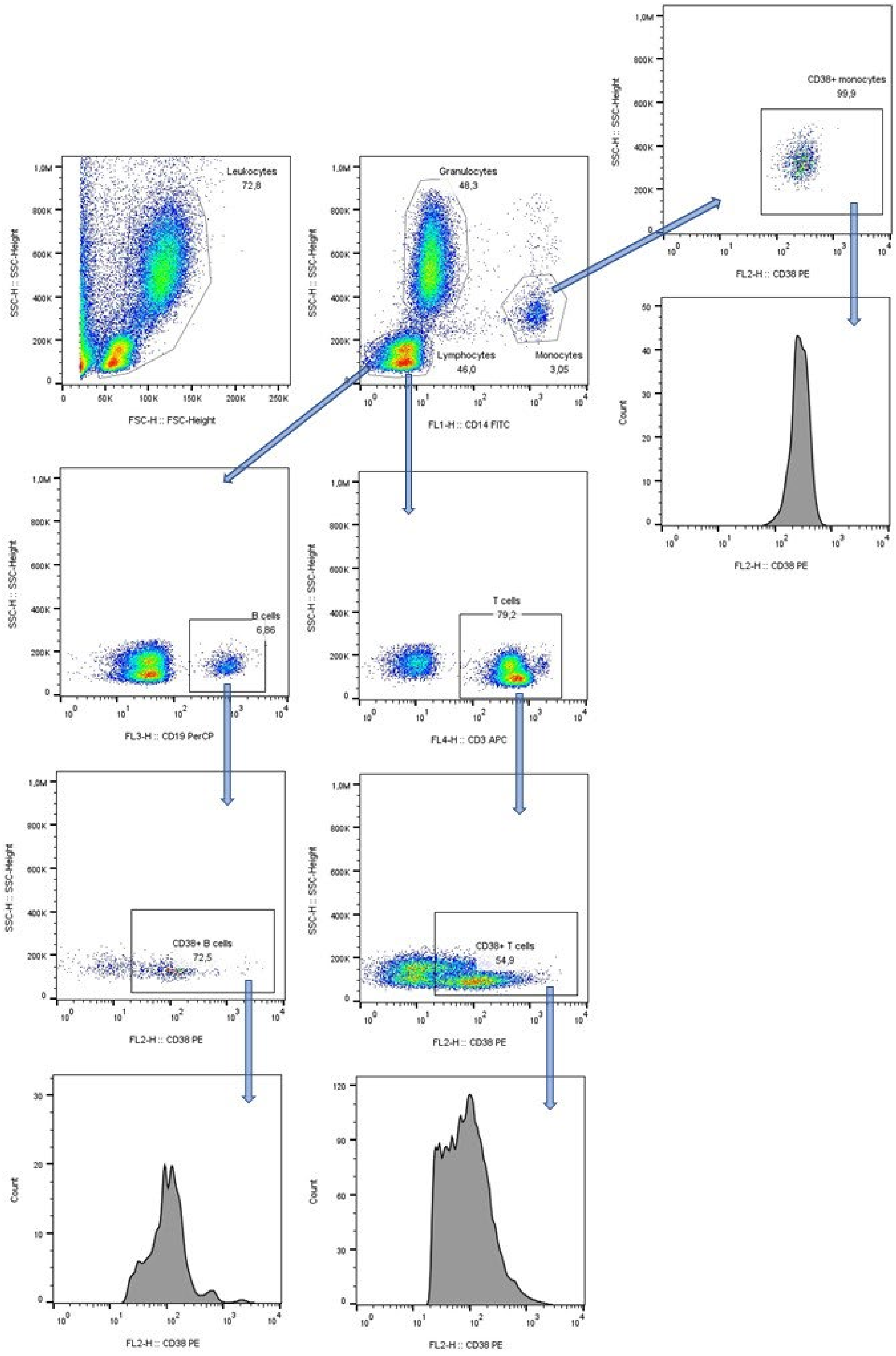

Figure S2. Flow cytometry gating strategy for leukocyte analysis of CD38. Intensity of the expression of CD38 was evaluated only in (sub)populations expressing CD38.

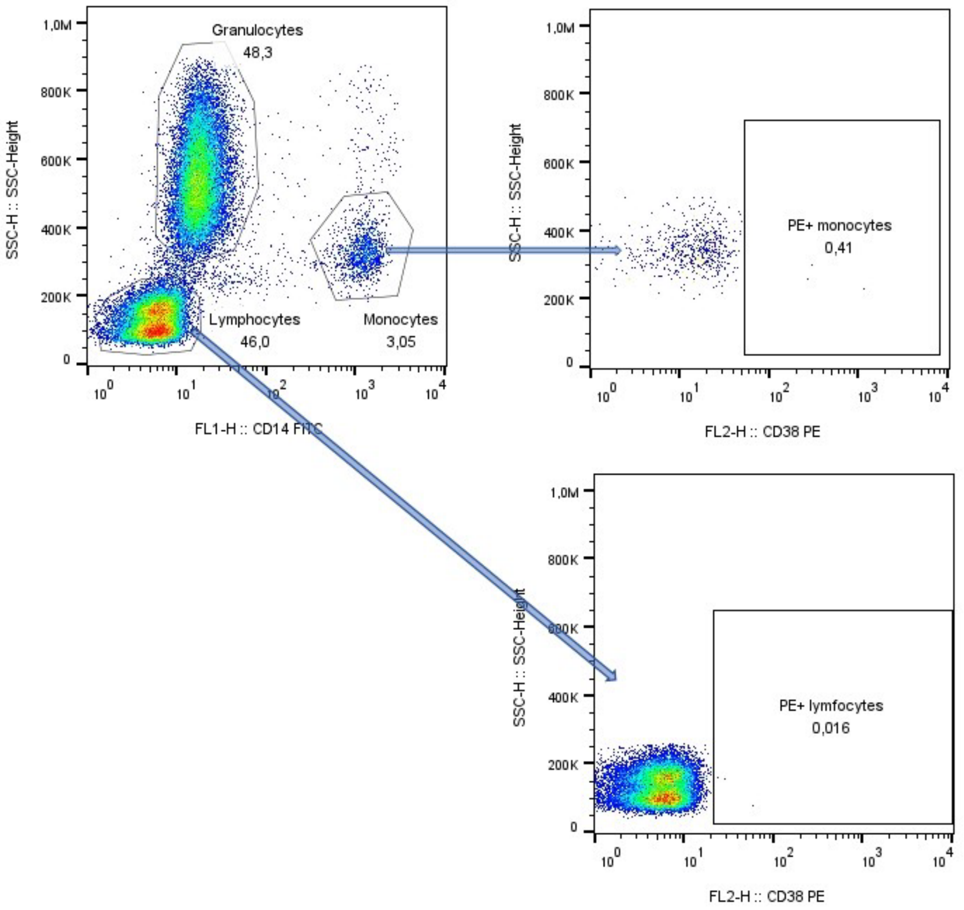

Figure S3. Setting for the positivity of CD38. Fluorescence minus one (FMO) was set using anti-CD3 APC, anti-CD19 PerCP, and anti-human CD14 FITC.

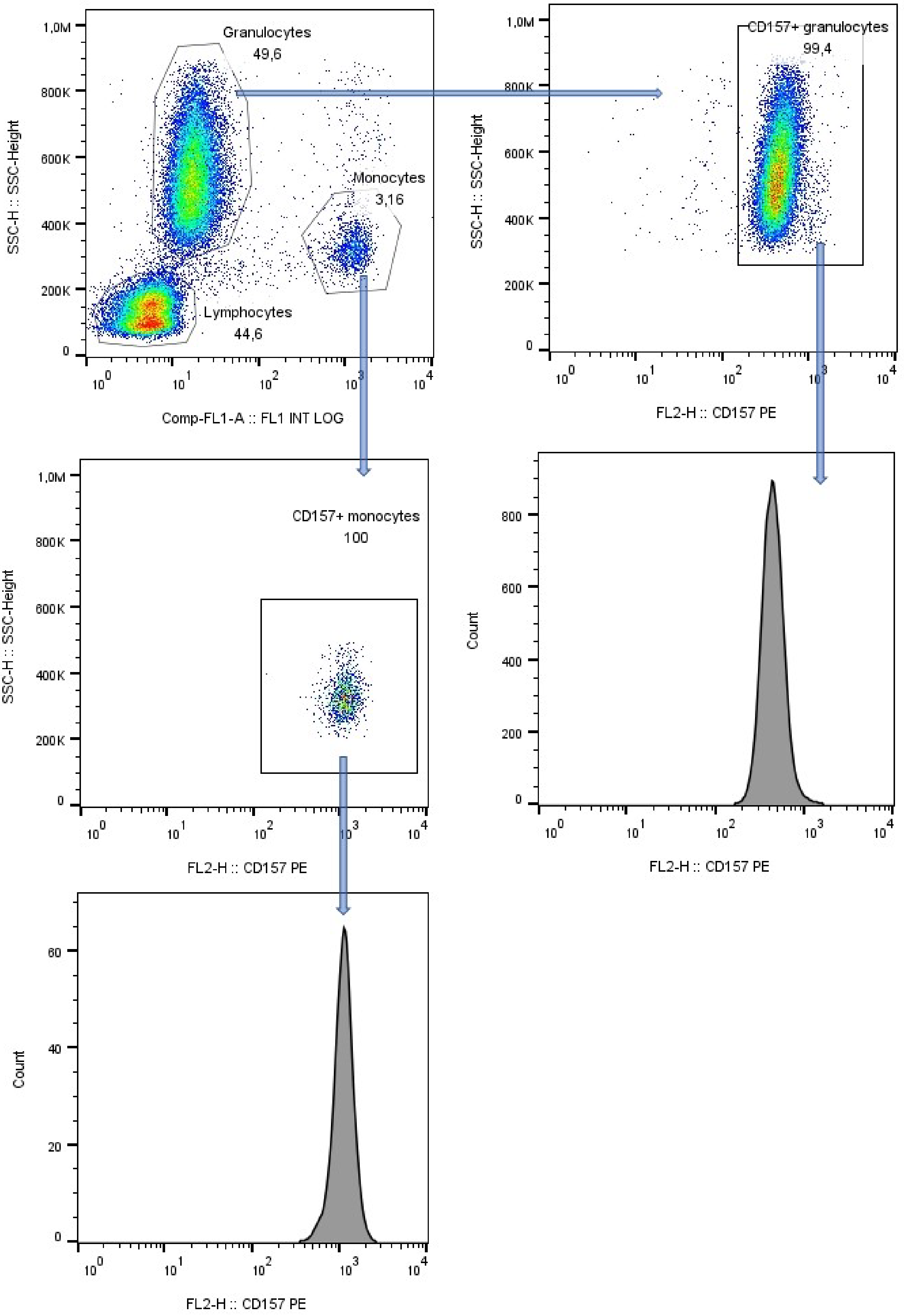

Figure S4. Flow cytometry gating strategy for leukocyte analysis of CD157. Intensity of the expression of CD157 was evaluated only in (sub)populations expressing CD157.

Table S1. Genders

	Controls (20)	Patients (40)
Men (4)	1	3
Women (56)	19	37

No difference between the frequency of men and women when comparing patients and controls, $p = 1$, Fisher's exact test.

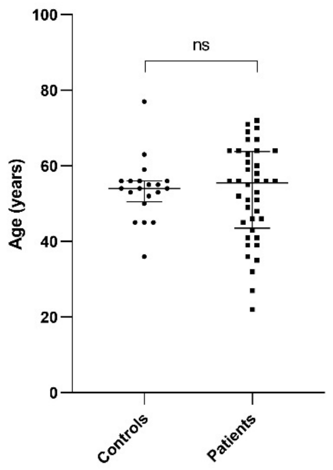

Figure S5. Comparison of the age of patients and controls, $p = 0.836$; ns: non-significant.
